# CCR5del32 genotype in human enteroviral cardiomyopathy leads to spontaneous virus clearance and improved outcome compared to wildtype CCR5

**DOI:** 10.1186/s12967-018-1610-8

**Published:** 2018-09-04

**Authors:** Dirk Lassner, Christine S. Siegismund, Uwe Kühl, Maria Rohde, Andrea Stroux, Felicitas Escher, Heinz-Peter Schultheiss

**Affiliations:** 1 Institute of Cardiac Diagnostics and Therapy (IKDT), Berlin, Germany; 2 Department of Cardiology, Campus Virchow, Charité-University Hospital Berlin, Berlin, Germany; 3 Institute of Biometry and Clinical Epidemiology, Campus Benjamin Franklin, Charité-University Hospital and Berlin Institute of Health, Berlin, Germany; 4 DZHK (German Centre for Cardiovascular Research), partner site Berlin, Berlin, Germany; 5 Berlin Institute of Health, Berlin, Germany

**Keywords:** Cardiomyopathy, CCR5del32 genotype, Coxsackievirus, Enterovirus, Interferon-beta therapy

## Abstract

**Background:**

Enteroviral cardiomyopathy is a life-threatening disease, and detection of enterovirus (EV) RNA in the initial endomyocardial biopsy is associated with adverse prognosis and increased mortality. Some patients with EV infection may spontaneously eliminate the virus and recover, whereas those with virus persistence deteriorate and progress to heart failure. Interferon-beta (IFN-β) therapy eliminates the virus, resulting in increased survival of treated patients. CCR5 is expressed on antigen-presenting cells (both macrophages and dendritic cells) and immune effector cells (T-lymphocytes with memory/effector phenotype and natural killer cells). Its 32-bp deletion (CCR5del32) is the most frequent human coding sequence mutation. This study addresses the correlation of CCR5 polymorphism to the clinical course of EV infection and the necessity for IFN-β treatment.

**Methods:**

We examined 97 consecutive patients with chronic/inflammatory cardiomyopathy and biopsy-proven EV infection and reliable information on clinical outcomes by CCr5 genotyping. These data were evaluated in relation to virus persistence in follow-up biopsies and survival rates over a 15-year period.

**Results:**

Genotyping revealed a strong correlation between the CCR5del32 genotype and spontaneous virus clearance with improved outcomes. All patients with CCR5del32 eliminated EV spontaneously and none of them died within the observed period. In the group of untreated CCR5 wildtype patients, 33% died (Kaplan–Meier log-rank p = 0.010). However, CCR5 wildtype individuals treated with IFN-β are more likely to survive than without therapy (Kaplan–Meier log-rank p = 0.004) in identical proportions to individuals with the CCR5del32 genotype.

**Conclusions:**

These data suggest that CCR5 genotyping is a novel predictive genetic marker for the clinical course of human EV cardiomyopathies. Hereby clinicians can identify those EV positive individuals who will eliminate the virus spontaneously based on CCR5 phenotype and those patients with CCR5 wildtype genotype who would be eligible for immediate antiviral IFN-β treatment to minimize irreversible cardiac damage.

**Electronic supplementary material:**

The online version of this article (10.1186/s12967-018-1610-8) contains supplementary material, which is available to authorized users.

## Background

Enteroviral cardiomyopathy is a life-threatening disease and varies from subclinical to fulminant forms. Detection of enterovirus (EV) RNA in the initial endomyocardial biopsy (EMB) is associated with adverse prognosis and increased mortality [1–6]. The three global phases (primary infection, immunological, and chronic phases) of EV infection are expressed differentially in affected patients [7]. We previously showed that some patients with EV infection may spontaneously eliminate the virus and recover, whereas those with virus persistence deteriorate and progress to heart failure [8, 9]. In a recently published clinical phase II study, those patients prone to persistence are eligible for immediate initiation of antiviral interferon-β (IFN-β) therapy. This treatment eliminates enterovirus efficiently and should be started immediately after EMB based diagnosis to prevent irreversible damage to the heart muscle cells [8–10].

The CC-chemokine receptor 5 (CCR5) plays a crucial role within the immune system and is expressed on antigen-presenting cells (both macrophages and dendritic cells) and immune effector cells (T-lymphocytes with memory/effector phenotype and natural killer cells) [11]. Natural ligands are macrophage inflammatory protein 1α (MIP-1α), MIP-1β, the protein “regulated upon activation normal T cell expressed and presumably secreted” (RANTES), and monocyte chemotactic protein 2 (MCP-2). The gene is adjacent to chromosome 3p21 and shares 70% amino acid sequence identity with CCR2. A 32-basepair (bp) deletion (del32) in the CCR5 gene between nucleotides 554–585 causes a frame shift after amino acid 184 and is the most frequent and most studied human CCR5 coding sequence mutation. CCR5del32 allelic frequencies vary substantially by geographic origin and range from 1% to more than 15% among Caucasians [11]. This CCR5del32 mutation leads to a deficiency of this receptor for various pro-inflammatory cytokines [12] and viruses [13]. The truncated protein has only four transmembrane domains and is not expressed on the cell surface. Since macrophage-tropic HIV strains use CCR5 as co-receptor for entry to human cells, homozygosity results in reduced susceptibility to these HIV strains [13, 14] and, in contrary, appears fatal in West Nile Virus infection [15]. Together with CXCR4, CCR5 is the most preferred target gene for the gene editing CRISPR/cas9 system to eliminate a possible entry side for HIV [16].

This polymorphism is also associated with an improved outcome in diabetes and coronary heart disease [17]. Cardiovascular diseases are the most common cause of death in Western European countries. The European Society of Cardiology (ESC) estimates that 12 million patients in Europe are suffering from heart failure; of these, two million are showing dilated cardiomyopathy (DCM) [18]. DCM often develops after myocardial viral infections or inflammation [4, 19]. The most relevant cardiotropic viruses are Erythrovirus (Parvovirus B19), Human Herpes Virus 6, Adenovirus and EV (mainly Coxsackievirus B3) [1, 2, 5, 9, 20, 21]. In recent studies, we showed for the first time that the CCR5del32 polymorphism is an independent genetic factor that influences the outcome in patients with clinically suspected myocarditis and DCM [22]. Following a revised definition of DCM, genetic predispositions (e.g. multiple mutations in the *titin* gene) alone and in combination with environmental factors, such as alcohol intake, pregnancy, or virus infections, are showing enhanced risk for the development of cardiomyopathies [23].

The following study addresses a correlation of the CCR5 polymorphism with the long-term clinical course of EV cardiomyopathy. We hypothesized that the CCR5del32 genotype is associated with a beneficial clinical outcome and a reduced risk for mortality in EV-positive patients. CCR5 genotype could be a predictive marker for long-term survival. In a translational approach, this biomarker might indicate those patients who will benefit from antiviral treatment with IFN-β [8, 9].

## Methods

### Patients

Similar to our recent study [9], we included 97 patients (mean age ± standard deviation 50.5 ± 13.8 years; 66 men) with biopsy-based baseline and follow-up information on the PCR confirmed course of enterovirus infection in correlation with CCR5 polymorphism and 15-year all-cause mortality (mean ± SD follow-up period 99 ± 55 months). Out of over 5000 analysed patient samples obtained between 1998 and 2013, only these 97 patients with EMB proven enterovirus infection and follow-up EMBs could be identified. All patients were showing symptoms of moderate to severe heart failure for > 6 month, including dyspnoea on exertion, weakness, fatigue, reduced physical capacity, or angina at rest and non-ischemic wall motion abnormalities. Patients with other co-morbidities such as coronary artery disease, hypertrophic or restrictive cardiomyopathies, right ventricular dysplasia, valvular diseases, a history of uncontrolled hypertension (> 170/95 mmHg), increased alcohol or drug uptake, renal failure, chronic obstructive pulmonary disease, or systemic and autoimmune diseases with known cardiac involvement that would explain left ventricular dysfunction were excluded through angiography, echocardiography, and laboratory counts. All patients had been examined by EMB for the presence of intramyocardial inflammation and cardiotropic viruses at first presentation and at a 6-month follow-up EMB for determining the course of EV infection [5, 9]. Compared to the initial report from 2012 [9], the time window of the retrospective analyses on mortality has been extended to 15 years and CCR5 genotype as an additional predictive marker has been taken into account. In addition, the number of included patients varied from the initial report due to availability of samples for CCR5 genotyping and cytokine analysis in serum.

Initially, three groups of patients were defined based on their disease outcome treatment according to former classification [9]: EV clearance for those who eliminated the EV spontaneously, EV persistence for those who were not able to clear the virus within 6 months by themselves, and EV + IFN-β for those with EV persistence for 6 months and who received IFN-β treatment resulting in virus clearance [10]. Selection of treated patients was as described previously [9]. In brief, in the interferon treatment group, the treatment started within 4 months (mean ± SD 2.3 ± 1.9 months) after virus-positive follow-up biopsy since a 6-month persisting virus is considered to be a chronic infection. Eight million units of IFN-β were administered every other day for 6 months in addition to constant heart failure medication [10]. Clinical parameters, medications, gender, and age did not differ significantly between patient groups. Occurrence of the endpoint (death) was determined through direct knowledge of the patient’s status, contact with family members, or inquiries at the registration office. All patients gave their written informed consent for data storage and evaluation. The study conformed to the principles outlined in the Declaration of Helsinki and was approved by the local ethics committees. Patients’ data were anonymized for analyses. The clinical data are depicted in Table 1.

**Table 1:** Baseline characteristics of EV positive patients

Clinical parameters	EV persistence (n = 23)	EV clearance (n = 42)	EV persistence + IFN-β (n = 32)	ANOVA p value
Age (years ± SD)	54 ± 14	47 ± 15	50 ± 13	0.198
Male [n (%)]	18 (78.3)	26 (66.7)	22 (62.9)	0.379
NYHA I/II/III/IV (%)	8.3/66.7/16.6/8.3	16.0/56.0/24.0/4.0	4.2/54.2/37.5/4.2	0.451
EF at baseline (% ± SD)	45 ± 19	52 ± 18	51 ± 19	0.293
Endpoint of death [n (%)]	11 (47.8)	4 (9.5)	0 (0)	0.001
LVEDD (mm ± SD)	59 ± 10	54 ± 11	58 ± 7	0.121
LVEDS (mm ± SD)	46 ± 13	42 ± 12	41 ± 11	0.467
Palpitations (%)	27.8	54.3	34.6	0.122
Arrhythmias (%)	26.7	46.7	25.0	0.219
AVB (%)	11.1	6.7	8.3	0.870
RBBB (%)	11.1	9.7	4.2	0.677
LBBB (%)	11.8	22.6	8.7	0.346
Syncopies (%)	23.5	11.4	3.8	0.146
Diabetes (%)	11.1	18.4	19.2	0.635
Glycosides (%)	30.0	12.5	40.7	0.061
Diuretics (%)	42.9	59.1	70.0	0.297
Β-blocker (%)	43.8	21.9	57.7	0.686
ACE (%)	70.6	56.7	70.8	0.483
Cumarin (%)	25.0	12.9	29.6	0.292
Antiarrhythmics (%)	30.8	13.6	15.8	0.435
Pacemaker/ICD (%)	10/10	0/2.8	0/5.8	0.146

*IFN-β* interferon-β, *NYHA* New York Heart Association Functional Classification, *EF* ejection fraction, *LVEDD* left ventricular end-diastolic diameter, *LVEDS* left ventricular end-systolic diameter, *AVB* atrioventricular block, *RBBB* right bundle branch block, *LBBB* left bundle branch block, *ACE* angiotensin-converting-enzyme, *ICD* implantable cardioverter-defibrillator

### EMB total RNA isolation, reverse transcription and nested-PCR for enteroviral RNA

Total RNAs were isolated during routine EMB diagnostics using Trizol reagent (Life Technologies, Darmstadt, Germany), treated with DNAse (PeqLab, Erlangen, Germany) to remove any traces of genomic DNA, and reverse-transcribed to cDNA with the High Capacity Kit (Life Technologies, Darmstadt, Germany) using random hexamers. Detection of enteroviral genomes by nested-PCR was performed as described elsewhere [4, 5].

### Genomic DNA isolation from PBMC and CCR5 genotyping

Genomic DNA from erythrocyte-lysed EDTA blood (1 mL) was extracted using the Puregene Mousetail Kit (Gentra, Minneapolis, MN, USA) according to the manufacturer’s protocol.

PCR was performed to detect the CCR5 polymorphism generating a PCR product of either 262 bp, 230 bp, or both lengths [22]. Isolated DNA was subjected to PCR amplification with primers for the CCR5 gene spanning the possible 32-bp deletion region on chromosome 3p21.31 (Accession No: NM_000579), forward primer HRF 5′-CTTCATCATCCTCCTGACAATCG-3′ and reverse primer HRR 5′-GACCAGCCCCAAGATGACTATC-3′. The reaction mixture was as follows: 2.5 µL 10× AmpliTaq buffer, 1 µL forward and 1 µL reverse primer, 4 µL 100 mM dNTPs, 0.25 µL 5 U/µL AmpliTaq enzyme, 13.75 µL aqua dest. to adjust the volume to 22.5 µL and 2.5 µL patient’s sample gDNA. PCR was carried out in an Eppendorf Mastercycler (Eppendorf, Hamburg, Germany), and the conditions were as follows: initial denaturation step of 7 min at 95 °C, 35 cycles of denaturation for 45 s at 95 °C, annealing for 45 s at 57 °C, and elongation for 45 s at 72 °C, followed by a final elongation for 10 min at 72 °C. PCR products were separated on an ethidium bromide-stained 2% agarose gel by electrophoresis and visualized by ultraviolet light. A PCR product of 262 bp in length indicated the wildtype CCR5 gene, 230 bp the homozygous CCR5 deletion of 32 bp, and both lengths showed the heterozygous phenotype (for details, see Additional file 1: Fig. S1).

### Multiplex measurement of serum cytokine levels

A total of 17 cytokines and chemokines in serum were measured by using the 17-plex Human Cytokine Panel Kit (Bio-Rad Laboratories, Inc. Hercules, CA, USA) at the time of first biopsy. The bead sets were analysed using a flow-based Luminex™ 100 suspension array system (Bio-Plex 100, Bio-Rad Laboratories). Sample cytokine concentrations were calculated by Bio-Plex Manager software (Bio-Rad Laboratories) using a five-parameter model of standard curves derived from the known reference cytokine concentrations supplied by the manufacturer.

### Statistical analysis

Descriptive statistics include absolute and relative frequencies for categorical variables and mean and standard deviation for quantitative measurements. Student’s *t*-test, Fisher’s exact test, one-way analysis of variance, Chi square and Kaplan–Meier with log-rank statistics were used for group comparisons, as appropriate. In detail, all Kaplan–Meier survival analyses were performed with use of time from sampling to death/census. Logistic regression was conducted to determine CCR5 genotype that independently distinguished between diagnostics groups. All probability values were two-tailed: p values below 0.05 were considered to indicate statistical significance, marked with asterisks for *p < 0.05 **p < 0.01 ***p < 0.001. No Bonferroni correction has been performed. IBM SPSS Statistics 23 (International Business Machines Corp, New York, NY, USA) was used to perform all statistical analyses and GraphPad Prism 4 (GraphPad Software Inc, La Jolla, CA, USA) software to generate all graphs.

## Results

In contrast to our initial report [9], the aim of this retrospective study was to evaluate the influence of the CCR5 genotype on viral persistence and associated mortality in patients with EMB proven EV positive cardiomyopathy over an extended time frame.

**Fig. 1:**
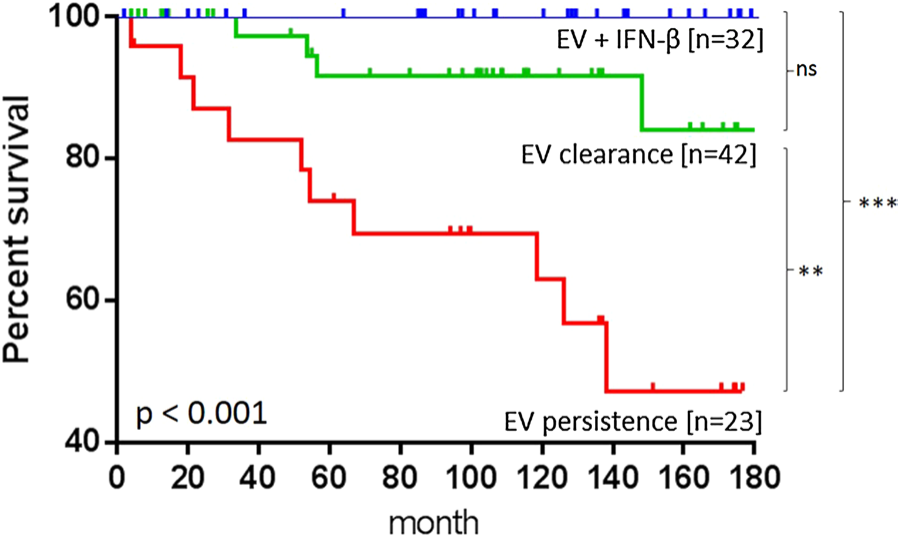
Survival proportions of EV patients grouped by their clinical course treatment. Long-term follow-up of patients with enteroviral cardiomyopathy revealed that cardiac EV persistence, due to the inability of a fraction of infected patients to eradicate the virus, is associated with a high mortality rate (red line). In contrast, patients capable of spontaneous virus elimination (as documented by a diagnostic EMB within approximately 6 months of initial presentation) or IFN-β therapy had a favourable long-term prognosis over 15 years (green line respectively blue line; Kaplan–Meier log rank statistics, p < 0.001). p values between the curves were indicates as *p < 0.05 **p < 0.01 ***p < 0.001 or ns for not significant

In these patients, we determined the CCR5 genotype by PCR in a series of patients (n = 97) with either spontaneous viral clearance (n = 42), persistence (n = 23), or IFN-β treatment (n = 32) confirmed by a follow-up biopsy and reliable information on the all-cause 15-year mortality. All three groups were not significantly different regarding clinical symptoms, hemodynamic or echocardiographic parameters at their initial presentation (Table 1). Significant difference (p = 0.001) was found for the endpoint of death (n = 15, 15.45% of all patients). Patients with spontaneous EV elimination or IFN-β treatment were significantly less likely to die (9.5%, n = 4 resp. 0%, n = 0) than patients with virus persistence (47.5%, n = 11) (red line, contingency Chi^2^ p < 0.001, Fig. 1). Under IFN-β treatment, all 32 patients cleared the enteroviral infection from the myocardium and all treated patients survived. At 15-year follow-up, the survival curves of these three groups were significantly different (Kaplan–Meier log rank p < 0.001). Patients with EV persistence (red line) had the poorest outcome compared to patients who spontaneously cleared EV (green line; p = 0.004) or who received IFN-β treatment (blue line; p < 0.001). The outcome of IFN-β treated patients (blue line) was considerably improved and comparable to patients with spontaneous virus clearance (green line; p < 0.092, Fig. 1).

In the next step, the same patients were reclassified into two new groups based on their CCR5 genotype: those homozygous for the major allele (wildtype, with functional receptor), and those with one or two deleted alleles (mutated, dysfunctional receptor). The percentage of the three CCR5 genotypes, in our patient cohort, is comparable with the published frequencies for the German population and for cardiac patients [wildtype (wt/wt; 79.4%), heterozygous (wt/del32; 18.6%), and homozygous for deletion (del32/del32; 2.1%)] [22, 24, 25]. Patients homo- or heterozygous for the deletion allele were clustered in one group (mutated) since the presence of one minor allele has already been associated with reduced receptor function [19, 23].

**Fig. 2:**
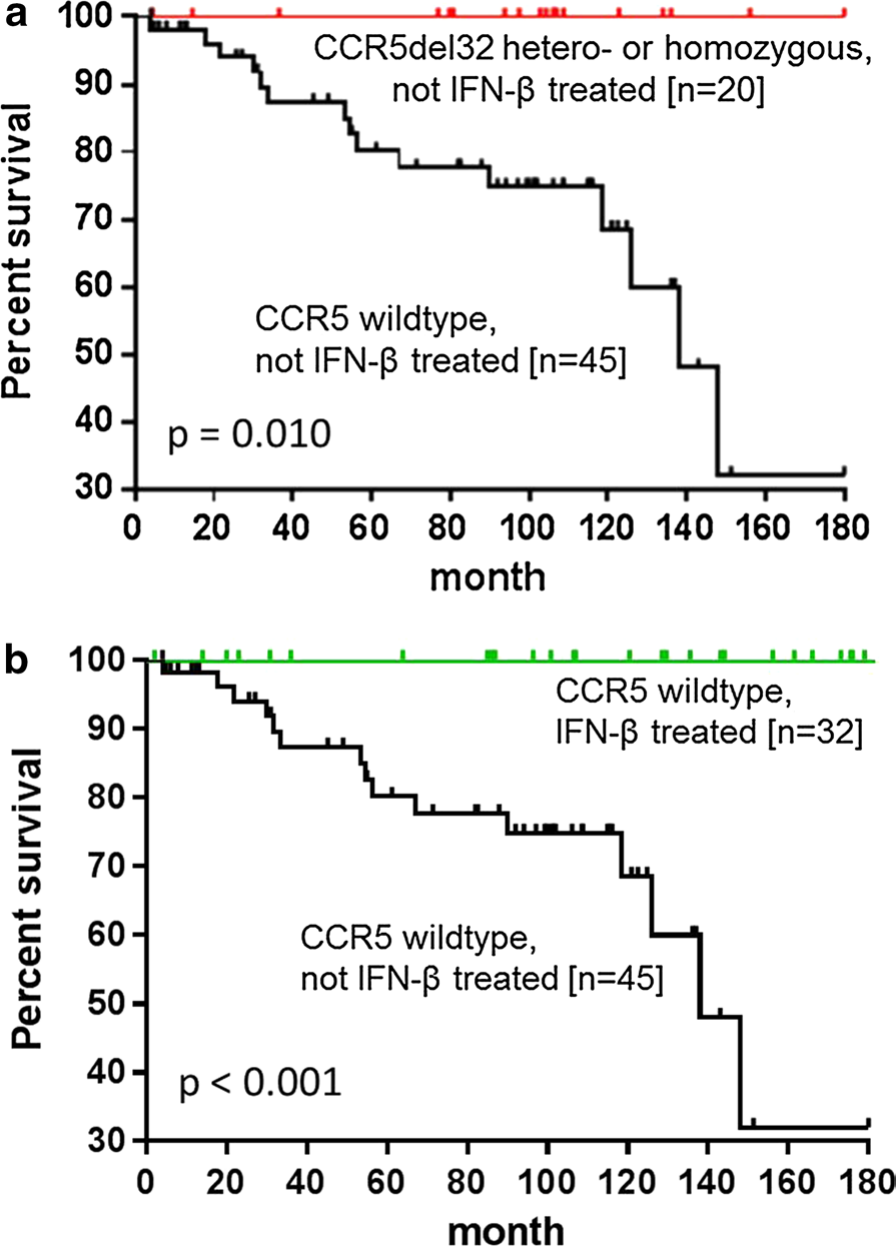
Survival proportions of EV patients grouped by their CCR5 genotype. At the 15-year follow-up, the overall mortality was 15.5% and the average period of follow-up from biopsy-based diagnosis was 102 ± 57 months (mean [± SD]) in our patient cohort (n = 97), classified by CCR5 genotyping. Survival curves (patients at risk) were generated according to the Kaplan–Meier method and compared with the log-rank statistic. **a** None of the patients with a 32-bp-deleted CCR5 died during a 15-year period (red line). In contrast, within the same observation period 15 out of 45 untreated individuals with the wildtype genotype met the endpoint of death. The survival rate with censored Kaplan–Meier calculation for EV positive patients without applied IFN-ß therapy was only about 33% in 15 years after initial EMB (Kaplan–Meier log-rank p = 0.010). **b** CCR5 wildtype individuals treated with IFN-β (green line) more likely to survive than without therapy (Kaplan–Meier log-rank p = 0.004) and reach survival proportions identical to individuals with the CCR5del32 genotype

CCR5 genotyping resulted in rearrangement of patients initially assigned into groups according to their clinical course of EV infection (Table 2). Interestingly, all patients with a CCR5del32 polymorphism (wt/del32, n = 18; and del32/del32, n = 2) eliminated the virus spontaneously and were alive at the end of the study (Kaplan–Meier log rank p = 0.010; Fig. 2a). Accordingly, CCR5 wildtype group comprised all patients with detectable virus persistence (29.8%, Table 2) and therefore they had a poorer clinical outcome. In the long-term survival analysis, all patients who met the endpoint of death (n = 15) were clustered in the group of 45 untreated CCR5 wildtype individuals. The tremendous effect of EV persistence on mortality is underlined by the fact that the majority of these individuals (n = 10 of 15) had died within the first 6 years after initial EMB. This effect is exceedingly increased with plotting the long-term survival by censor-based, time-adjusted Kaplan–Meier calculation [26, 27]. At the end of the 15-year period, only 33% of all CCR5 wildtype patients with EV persistence without specific treatment survived.

**Table 2:** Baseline characteristics of EV-positive patients grouped by their CCR5 genotype

	CCR5del32 hetero- or homozygous (n = 20)	CCR5 wildtype (n = 77)	p-value
Clinical classification*			
EV persistence [n (%)]	0 (0.0)	23 (29.8)	0.003
EV clearance [n (%)]	20 (100.0)	22 (28.5)	< 0.001
EV + IFN [n (%)]	0 (0.0)	32 (41.5)	< 0.001
Endpoint of death [n (%)]^a^	0 (0.0)	15 (19.48)	0.036
Clinical baseline parameters^#^			
Age (years ± SD)	49 ± 13	51 ± 14	0.541
Male [n (%)]	14 (70.0)	52 (67.5)	0.831
NYHA I/II/III/IV (%)	30/50/10/10	10/52/36/2	0.237
EF at baseline (% ± SD)	62 ± 12	48 ± 19	0.006
LVEDD (mm ± SD)	51 ± 9	57 ± 10	0.022
LVEDS (mm ± SD)	41 ± 13	44 ± 12	0.387
Palpitations (%)	66.7	50.1	0.390
Arrhythmias (%)	42.9	36.4	0.747
AVB (%)	0.0	10.4	0.348
RBBB (%)	12.5	4.3	0.352
LBBB (%)	25.0	12.8	0.374
Syncopies (%)	11.1	9.4	0.878
Diabetes (%)	20.0	15.5	0.727
Glycosides (%)	25.0	21.2	0.776
Diuretics (%)	62.5	63.9	0.920
Β-blocker (%)	53.8	66.0	0.630
ACE (%)	50.0	76.0	0.077
Cumarin (%)	8.3	22.0	0.667
Antiarrhythmics (%)	0.0	18.8	0.193
Pacemaker/ICD (%)	0.0/0.0	6.1/2.0	0.425

*IFN-β* interferon-β, *NYHA* New York Heart Association Functional Classification, *EF* ejection fraction, *LVEDD* left ventricular end-diastolic diameter, *LVEDS* left ventricular end-systolic diameter, *AVB* atrioventricular block, *RBBB* right bundle branch block, *LBBB* left bundle branch block, *ACE* angiotensin-converting-enzyme, *ICD* implantable cardioverter-defibrillator

Statistical analysis for p-values by *Fishers’s exact test and ^#^Student’s t-test

^a^All patients who met the endpoint of death had persisting enterovirus and no IFN-β therapy

In contrast, no CCR5 wildtype patient met the endpoint of death by applying an IFN-β therapy for 6 months. This antiviral treatment improved the all-cause mortality to a level identical to the survival curve of patients with the CCR5del32 genotype (Kaplan–Meier log rank p < 0.001; Fig. 2b).

The reduced mortality (Kaplan–Meier log rank p = 0.010) of CCR5del32 patients compared with wildtype individuals without IFN-β treatment might indicate that the CCR5del32 polymorphism is a protective factor in patients with EV cardiomyopathies (log regression p = 0.002, Fig. 2a). This finding is supported by the arrangement of clinical data according to CCR5 genotype, which showed that ejection fraction (EF) and related left ventricular end-diastolic diameter (LVEDD) are significantly different between patients with the wildtype and CCR5del32 genotype at the time of initial biopsy [EF (mean ± SD): CCR5wt 48 ± 19% vs. CCR5del32 62 ± 12%; LVEDD (mean ± SD): CCR5wt 57 ± 10 mm vs. CCR5del32 51 ± 9 mm; Student’s t-test p = 0.006; Table 2]. The patient cohort showed no significant differences between groups or within one group regarding clinical symptoms, hemodynamic parameters, or echocardiographic parameters at their initial presentation whereas EF and LVEDD are significantly improved in patients with CCR5del32. The CCR5del32 genotype not only distinguishes patients with spontaneous virus elimination from patients with persistence but these patients also show better initial conditions in the natural course of EV infection.

The baseline serum levels of 17 human cytokines were determined to elucidate a potential influence of cytokines on the clinical course of EV positive patients. No statistical differences are shown either between all three clinical groups or between EV persistence and clearance, IFN-ß treatment (for details, Table 3). Reanalysing serum cytokine levels within the new reclassified groups shows no significant differences between CCR5 wildtype and CCR5del32 phenotype individuals either (Table 4) [9].

**Table 3:** Baseline serum cytokine levels grouped by their clinical course resp. treatment

Serum cytokine levels (mean ± SD, pg/mL)	EV persistence (n = 23)	EV clearance (n = 42)	EV persistence + IFN-β (n = 32)	ANOVA p value all 3 groups	Student’s *t*-test p value persistence vs. clearance
IL-1β	3.90 ± 7.61	6.72 ± 12.72	1.41 ± 3.20	0.174	0.405
IL-4	1.03 ± 2.99	3.43 ± 11.51	0.89 ± 1.97	0.448	0.393
IL-5	0.52 ± 0.51	0.98 ± 2.93	1.76 ± 3.30	0.347	0.521
IL-6	55.46 ± 64.38	58.57 ± 96.80	35.25 ± 30.62	0.530	0.906
IL-7	1.55 ± 1.31	4.22 ± 8.76	3.33 ± 3.77	0.356	0.208
IL-8	9.70 ± 11.11	5.63 ± 8.68	3.98 ± 3.01	0.099	0.180
IL-10	3.81 ± 2.85	6.38 ± 11.33	6.98 ± 10.08	0.557	0.365
IL-12 (p70)	1.31 ± 2.51	4.27 ± 10.01	10.81 ± 39.17	0.430	0.228
IL-13	1.09 ± 1.64	10.67 ± 48.17	2.26 ± 5.01	0.525	0.406
IL-17	4.07 ± 6.11	3.86 ± 8.21	2.69 ± 5.17	0.787	0.927
G-CSF	13.91 ± 14.63	17.78 ± 28.28	9.70 ± 8.95	0.413	0.597
GM-CSF	24.96 ± 38.32	71.73 ± 140.00	22.61 ± 33.09	0.339	0.314
IFNγ	63.81 ± 182.41	49.97 ± 87.50	11.9 ± 13.14	0.386	0.485
MCP-1 (MCAF)	42.90 ± 66.10	67.85 ± 163.90	24.55 ± 10.85	0.418	0.545
MIP-1β	37.66 ± 28.71	42.56 ± 55.97	30.30 ± 12.75	0.583	0.735
TNFα	5.81 ± 14.98	8.57 ± 17.14	5.22 ± 13.03	0.740	0.589

*IL* interleukin, *G-CSF* granulocyte-colony stimulating factor, *GM-CSF* granulocyte–macrophage colony-stimulating factor, *IFN* interferon, *MCP* monocyte chemo attractant protein, *MIP* macrophage inflammatory protein, *TNF* tumour necrosis factor

**Table 4:** Baseline serum cytokine levels in EV patients grouped by their CCR5 genotype

Cytokine serum levels (mean ± SD, pg/mL)	CCR5del32 hetero- or homozygous (n = 20)	CCR5 wildtype (n = 77)	Student’s t-test p value
IL1b	3.39 ± 7.35	8.18 ± 19.64	0.425
IL4	0.42 ± 0.74	6.68 ± 31.56	0.486
IL5	0.58 ± 1.04	1.08 ± 2.40	0.487
IL6	23.62 ± 19.20	132.50 ± 512.10	0.455
IL7	2.56 ± 2.03	4.08 ± 8.27	0.530
IL8	3.49 ± 2.86	7.27 ± 10.44	0.213
IL10	2.70 ± 3.38	12.56 ± 52.23	0.507
IL12p70	1.58 ± 2.34	11.17 ± 37.54	0.371
IL13	1.3 ± 1.92	7.05 ± 35.54	0.570
IL17	3.53 ± 3.71	4.49 ± 9.37	0.730
G-CSF	13.15 ± 16.06	27.25 ± 89.40	0.585
GM-CSF	75.12 ± 184.09	128.50 ± 563.16	0.546
IFNγ	22.10 ± 29.66	105.45 ± 463.46	0.415
MCP-1 (MCAF)	57.10 ± 89.03	48.61 ± 119.35	0.792
MIP-1β	41.06 ± 27.16	43.39 ± 62.13	0.892
TNFα	4.70 ± 6.26	10.80 ± 29.76	0.478

*IL* interleukin, *G-CSF* granulocyte-colony stimulating factor, *GM-CSF* granulocyte–macrophage colony-stimulating factor, *IFN* interferon, *MCP* monocyte chemo attractant protein, *MIP* macrophage inflammatory protein, *TNF* tumour necrosis factor

The very fact that individuals who received IFN-β treatment reached survival curves identical to survival curves of CCR5del32 individuals highlights IFN-β as an adequate therapy to overcome genetic differences (Fig. 2b). This significant improvement by IFN-β treatment emphasizes the need to start treatment as early as possible to prevent irreversible cardiac injury and adverse long-term prognosis due to cardiac persistent EV.

**Fig. 3:**
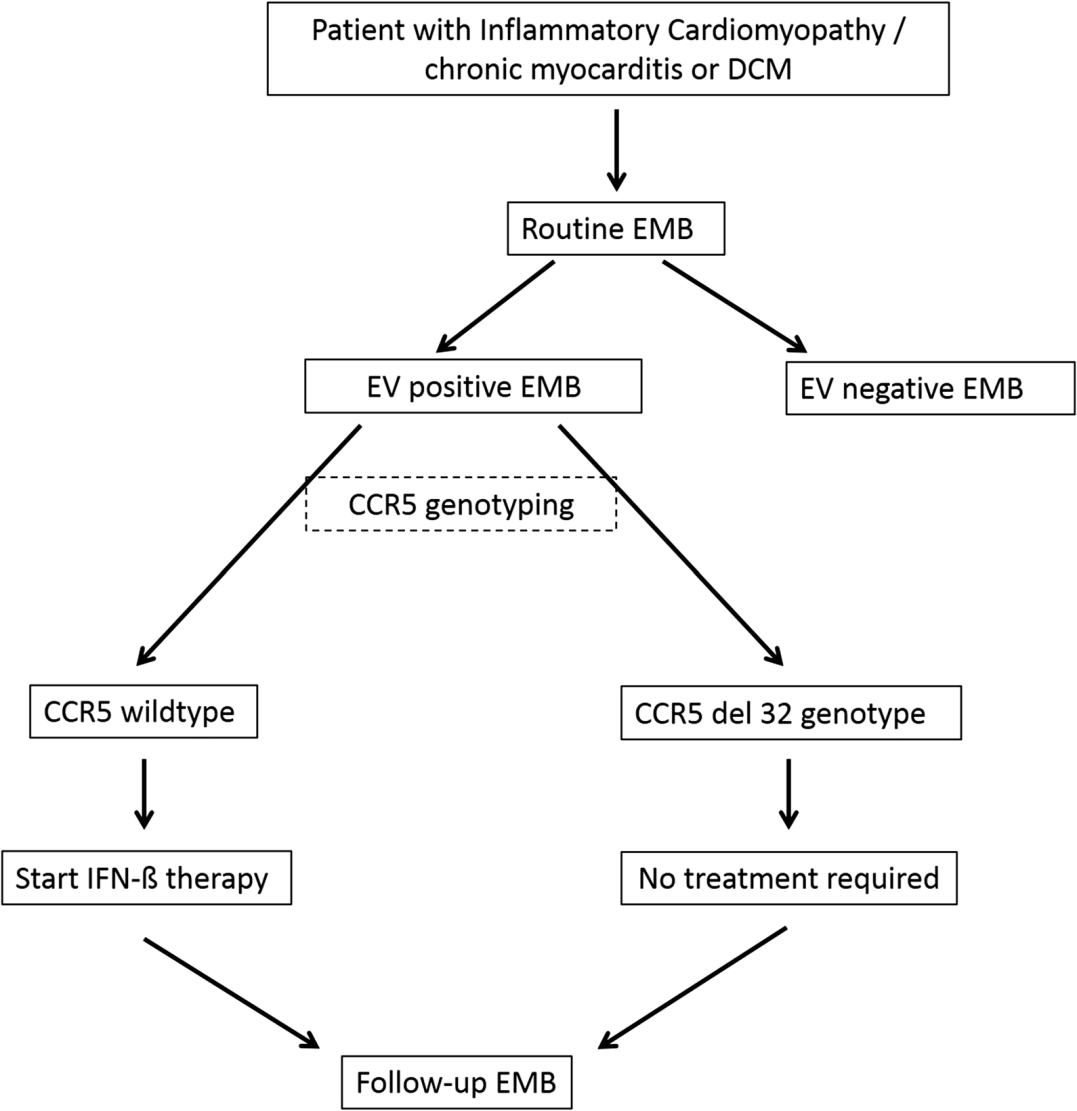
Proposal for initiation of IFN-ß therapy of EV-positive patients. At time point of EV positive EMB the patients should be genotyped for CCR5 mutation. If CCR5del32 genotype is detected, no treatment with IFN-β is required, only clinical monitoring. If CCR5 wildtype genotype is determined IFN-β treatment is recommended for elimination of EV and resulting in improvement of the clinical course

The translational value of the current study is based on giving recommendations for CCR5 genotyping for all patients with EV positive EMB. We propose a diagnostic scheme including genotyping resulting in the initiation of IFN-ß therapy for CCR5 wildtype patients as early as possible to prevent myocardial injuries (Fig. 3).

## Discussion

The present retrospective study revealed that the CCR5 polymorphism could predict the clinical course of myocardial EV infection shown by analysis of initial and follow-up EMBs. Genotyping demonstrated a clear allocation of patients into two genetically different patient groups with different clinical outcomes. The CCR5del32 genotype (heterozygous or homozygous) is clearly associated with complete spontaneous elimination of myocardial EV. All these individuals were alive at the end of this study, whereas a significant proportion (33.3%) of patients with CCR5 wildtype had died within a 15-year period.

Enteroviral RNA persistence is associated with progression of LV dysfunction and a lack of clinical improvement. At the 15-year follow-up, the patients with virus persistence had an increased mortality compared to patients who cleared the virus spontaneously [5, 9, 18]. In different pilot studies [16, 18] and in the recently published clinical phase II trial [10], persisting EV infection was treated by IFN-ß resulting in EV elimination, clinical improvement, and significantly reduced mortality.

CCR5 genotyping has the potential as a predictive marker to identify individuals with myocardial EV infection who would benefit from antiviral treatment to overcome progression of the disease and increase their survival rate. In our recent study [9], and also in this current study, it was shown that IFN-ß therapy eliminates EV and no treated patient died. Nevertheless, the treatment with IFN-ß over 6 months is associated with multiple side effects such as flu-like symptoms, haematological toxicity, elevated transaminases, nausea, fatigue, and psychiatric sequelae [28] which often lead to termination and interruption of therapy. CCR5 genotyping allows the identification of patients who would benefit from IFN-β therapy and, simultaneously, the identification of patients who would not benefit and would therefore need to deal unnecessarily with exhausting medication.

The coincidence of a genetic predisposition with an environmental factor could influence the clinical follow-up of affected patients dramatically [23]. The influence of a genetic variation on human enteroviral cardiomyopathy was shown in detail for the Toll-like receptor 3 (TLR3) gene which mediates the innate antiviral response in myocardium [29]. Mutations in this gene affect the host’s susceptibility to enteroviral cardiomyopathies by inhibition of NF-kB and type 1 interferon pathway. In cell lines expressing the mutated TLR 3, the Coxsackievirus replication was increased significantly, resulting in reduced viral clearance [30].

CCR5 is crucial for the antiviral immune response and closely regulated by IRAK4, which suppresses several antiviral key mechanisms [31]. Loss of IRAK 4 function in knockout mice increases the number of CCR5 on monocytes and leads to the elimination of enterovirus and better survival of enterovirus-infected mice. In this mouse model, the infiltration of CCR5 + monocytes/macrophages is beneficial for virus elimination and therefore shows controversial results to our study of humans [31].

IRAK and TLR3 influence the production of type 1 interferons and pro-inflammatory cytokines [30]. Serum IFN-ß levels have previously been shown to be significantly elevated in patients who cleared the virus spontaneously in comparison with patients having virus persistence [9, 19]. Since the presence of one minor allele has already been associated with reduced receptor function in binding its ligands MIP-1α, MIP-1β, RANTES, and MCP-2 [32], a deletion in the CCR5 gene implies an involvement of immune system components as shown for impaired macrophage and leukocyte infiltration [33, 34], systemic inflammation [12], and the evolutionary pro-inflammatory response [35]. CCR5 is not the unique receptor for its natural ligands, which also use CCR1 [36, 37]. In addition, MCP-2 also binds to CCR2B [37] and MIP-1α resp. RANTES also interacts with CCR4 [38]. Although chemokine receptors are not equally exchangeable [15], cytokine levels must not be affected by mutations in this particular receptor and thus would explain similar cytokine levels of all groups in our EV study cohort. This effect does not exclude an influence of mutated CCR5 to the immune system but emphasizes its status as an independent genetic factor. Further investigations are required to reveal the molecular pathomechanism in humans and mice to overcome divergences of experimental mouse models with clinical courses of human patients.

The importance of the CCR5 genotype for viral infections was demonstrated impressively by the cure of HIV infection by transplantation of CCR5del32 homozygous stem cells [13, 14]. In contrast, hepatitis C patients who are homozygous for the CCR5del32 deletion have been reported to carry increased viral loads [34]. The increased prevalence of CCR5del32 homozygosity associated with increased viral loads in patients with chronic hepatitis C suggests that the CCR5del32 mutation may be an adverse host factor in hepatitis C. Only CCR2 has been reported as underrepresented in HCV patients who cleared the virus spontaneously, and in that study, none of the other variants in the CCR gene cluster, such as CCR1 and CCR5, showed association with the natural course of the infection, stage of fibrosis, or response to therapy [33].

Whereas homozygosity is necessary for affecting the host’s response in HIV and HCV, in severe malaria heterozygous, deficiency for G6PD gives advantages and the homozygous leads to disadvantages [39]. Interestingly, an effect on EV clearance and mortality is already shown for heterozygous mutation of the CCR5 receptor in our cohort of 97 patients, which is the largest enteroviral cardiomyopathy cohort ever reported about [9].

As shown in a former study, CCR5del32 is a positive prognostic marker for diabetes and survival in patients with different forms of virally-induced or inflammatory cardiomyopathies [22]. The clinical or molecular reason for this improved clinical outcome is still unknown. Generally, a higher EF in heart muscle diseases is associated with a better prognosis. Therefore, the initially higher EF of the CCR5del32 patients compared to CCR5 wildtype individuals could be one cause for a better outcome. The underlying molecular mechanism of the CCR5 tropism, resulting in better initial conditions of patients, needs to be investigated in a larger cohort with functional analyses. In consequence, low EF and increased LVEDD additionally burdens the patients. Only CCR5 genotyping is a suitable prognostic marker to predict outcome and therapy decision for EV positive patients.

The CCR5 genotyping has the advantage of identifying those patients who would benefit from antiviral treatment by the determination of a single easily accessible genomic marker. The application of proposed genotyping allows a clear-cut differentiation between patients with spontaneous cleared or persisting EV cardiomyopathy and enables cardiologists to individualize patient-associated treatment decisions as well as initiate a disease-directed antiviral therapy in patients with CCR5 wildtype. The application of IFN-β results in the elimination of EV and improved survival curves identical to curves of patients with CCR5del32. This emphasises that the removal of any environmental factor minimises the burden of a genetic predisposition [23].

## Limitations

Thus far, this study is the largest retrospective analysis of cardiac patients with EV-induced cardiomyopathy in correlation to CCR5 polymorphism and 15-year all-cause mortality. The geographic distribution of study sites throughout Germany including mostly Caucasians might limit the possibility to transfer study results to other ethnicities and countries. Genotyping of different markers would be more informative and would possibly reveal a combination of markers to be best for prediction, but a single genomic marker was sufficient to separate patients with spontaneous virus elimination from the group of patients with EV persistence. Due to the limited size of our dataset, the use of genotyping to guide therapy and the necessity to start IFN-therapy as early as possible needs to be validated in prospective, randomized, multicentre trials.

## Conclusion

Our data show that CCR5del32 polymorphism is a predictive factor for persistence and clearance of myocardial EV and facilitates therapy decisions for a defined group of infected patients. The underlying pathomechanism of the CCR5 polymorphism on distinct outcomes in heart muscle diseases is not fully understood and requires further investigations.

Antiviral IFN-β therapy eliminates the virus, resulting in an increased survival of treated patients but should only be administered to patients who would benefit, due to side effects [8, 10]. The proposed genotyping of a 32-basepair deletion in the CCR5 gene has the predictive power to identify patients with suspected EV persistence who would benefit from an immediate application of IFN-ß treatment. Simultaneously, it would prevent patients with good clinical prognosis from unnecessary stressful treatment based on intrinsic predisposition to eliminate the virus spontaneously. This diagnostic work-up is in complete accordance with the current proposal of ESC working group on myocardial and pericardial diseases for an aetiology oriented approach to tailored therapy in acquired cardiomyopathies with genetic predisposition [23]. Genotyping and EMB enables cardiologists to individualize patient-associated treatment decisions and to initiate a disease-directed antiviral therapy in patients with the CCR5 wildtype genotype.

## Additional file

**Additional file 1: Fig. S1.** CCR5 genotyping PCR. **a** Agarose gel electrophoresis (2%) showing DNA lenghts marker (9 DNA fragments with different lenghts) and 16 lanes with PCR products generated from patient samples: a 260bp product for wt/wt, two PCR products with a lengths of 260 bp and 230 bp for heterozygous CCR5del32/wt and a PCR product with 230 bp in length for homozygous CCR5del32/CCR5del32. **b** Sequence of the 262 bp PCR product of the genotyping PCR indicating the CCR5del32 deletion in red and the primer-binding sites with arrows.

## Abbreviations

CCR5: CC-chemokine receptor 5
CCR5del32: CCR5 32-basepair deletion
DCM: dilated cardiomyopathy
EMB: endomyocardial biopsy
EV: enterovirus
EF: ejection fraction
HCV: hepatitis C virus
HIV: human immunodeficiency virus
IFN-β: interferon-β
LVEDD: left ventricular end-diastolic diameter
LVESD: left ventricular end-systolic diameter
MIP-1: macrophage inflammatory protein 1
MCP-2: monocyte chemotactic protein 2
PBMC: peripheral blood mononuclear cell
PCR: polymerase chain reaction
RANTES: regulated upon activation normal T cell expressed and presumably secreted
RT-PCR: reverse transcription PCR
wt: wildtype
