# Supplementary figures and images for: CCR5del32 genotype in human enteroviral cardiomyopathy leads to spontaneous virus clearance and improved outcome compared to wildtype CCR5
Source: J Transl Med. 2018 Sep 4;16:249. doi: 10.1186/s12967-018-1610-8 (PMC6123922; doi:10.1186/s12967-018-1610-8)

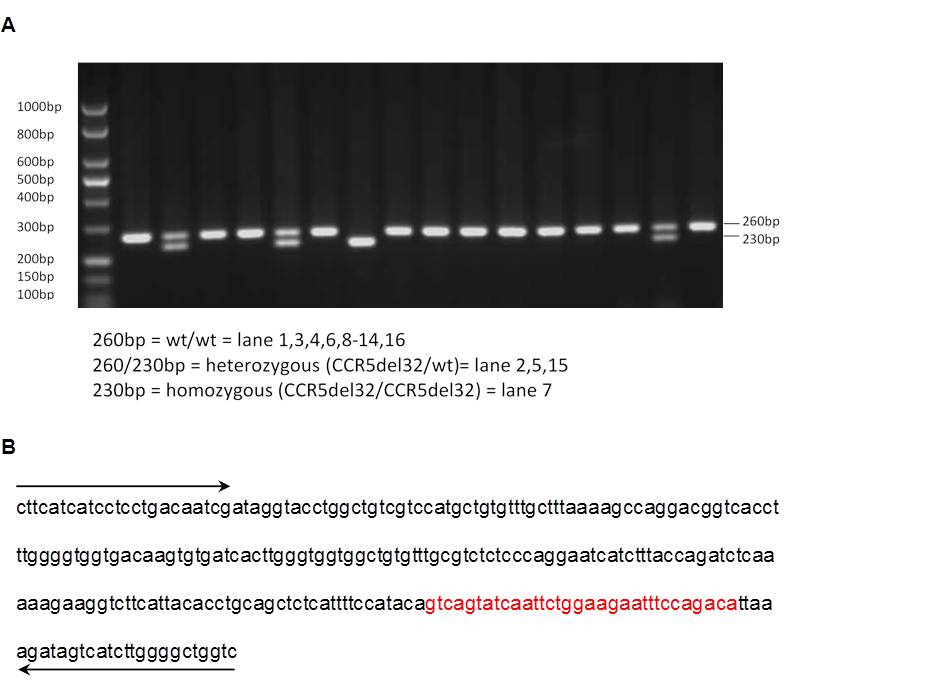

Supplement: Supplementary file 1 — Additional file 1: Fig. S1. CCR5 genotyping PCR. a Agarose gel electrophoresis (2%) showing DNA lenghts marker (9 DNA fragments with different lenghts) and 16 lanes with PCR products generated from patient samples: a 260bp product for wt/wt, two PCR products with a lengths of 260 bp and 230 bp for heterozygous CCR5del32/wt and a PCR product with 230 bp in length for homozygous CCR5del32/CCR5del32. b Sequence of the 262 bp PCR product of the genotyping PCR indicating the CCR5del32 deletion in red and the primer-binding sites with arrows. [file 12967_2018_1610_MOESM1_ESM.jpg]
